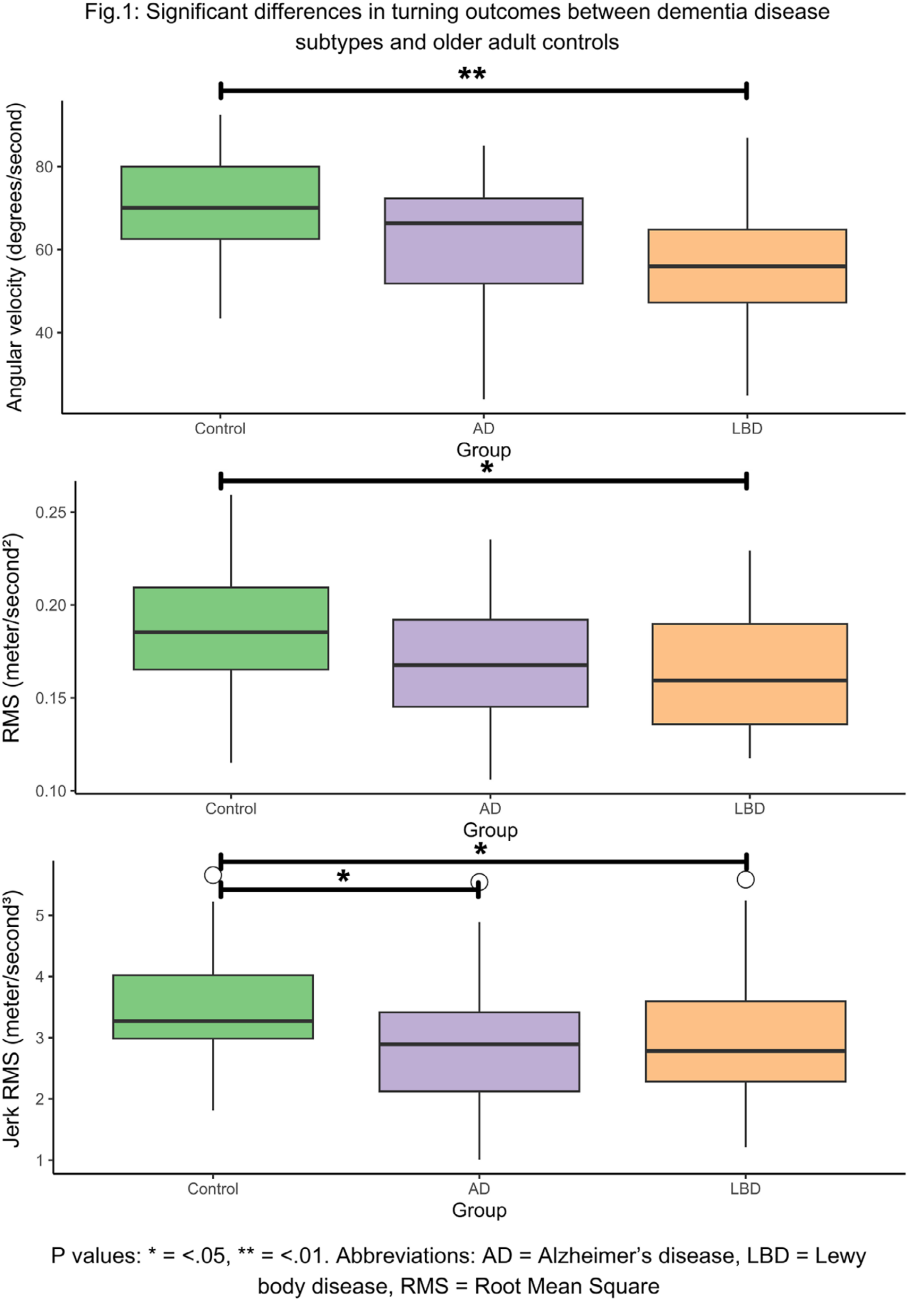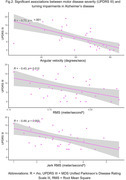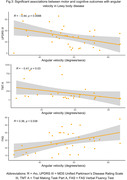# Digital assessment of turning behaviours in Alzheimer’s disease and Lewy body disease and their association with cognitive function

**DOI:** 10.1002/alz.092231

**Published:** 2025-01-09

**Authors:** Ríona Mc Ardle, Silvia Del Din, Rana Zia Rehman, Brook Galna, Alan J Thomas, Lynn Rochester, Lisa Alcock

**Affiliations:** ^1^ Newcastle University, Newcastle Upon Tyne UK; ^2^ Newcastle University, Newcastle, Tyne and Wear UK; ^3^ Centre for Healthy Ageing, Murdoch University, Murdoch, Perth, Western Australia Australia; ^4^ Murdoch University, Perth, Western Australia Australia; ^5^ Newcastle University, Newcastle upon Tyne UK; ^6^ Newcastle University, Translational And Clinical Research Institute, Newcastle upon Tyne UK

## Abstract

**Background:**

Digital mobility outcomes (DMOs) can be captured using body‐worn inertial measurement units (IMUs) in lab‐based and real‐world environments. DMOs may support differential diagnosis of dementia; for example, Alzheimer’s disease (AD) and Lewy body disease (LBD) show unique signatures of gait impairment. Growing evidence suggests that turning impairments are related to cognitive decline. Turning outcomes can be captured via IMUs and may complement use of existing DMOs. This study aims to assess differences in turning outcomes between AD, LBD and older adult controls, and explore their clinical and cognitive correlations.

**Method:**

Participants completed six intermittent 10‐metre walks while wearing a IMU (APDM, Opal 128Hz) attached to their lower back. Following each walk, they made a 180degree turn. Two spatiotemporal (turn duration, angular velocity) and two acceleration‐based (combined direction [anterior‐posterior/medio‐lateral/vertical] Root Mean Square (RMS) and Jerk RMS) turning outcomes were extracted using validated algorithms. Analysis of covariance assessed between‐group differences in turning outcomes, controlling for age and sex. Tukey HSD tests for multiple comparisons determined where differences occurred. Correlations (Spearman’s rho) between significant turning outcomes and age, motor disease severity (MDS Unified Parkinson’s Disease Rating Scale III), global cognition (standardised Mini Mental State Examination; sMMSE), information processing (Trail Making Task A), and executive function (FAS Verbal Fluency Test) were assessed.

**Results:**

98 eligible participants from the GaitDem study were included: 28 controls (Age (mean±SD):74±9 years, sMMSE:29±1), 35 AD (Age:77±6, sMMSE:23±4), and 35 LBD (Age:76±6, sMMSE:24±3). Both dementia disease subtypes demonstrated lower jerk RMS compared to controls (p<.05), while people with LBD also showed lower angular velocity, RMS and Jerk RMS (p<.05; Figure 1). For AD, turning impairments were associated with greater motor disease severity (Figure 2). For LBD, slower angular velocity was associated with greater motor disease severity, slower information processing and worse executive function (Figure 3).

**Conclusions:**

Results suggest that dementia disease subtypes have significant turning impairments compared to normal ageing, but not compared to each other. However, turning impairments were related to discrete motor‐cognitive profiles in dementia disease subtypes. Further work should explore other turning characteristics (i.e. related to turn phase, signal direction, turn strategy) as differential markers of dementia.